# Nanofilament-Coated Superhydrophobic Membranes Show Enhanced Flux and Fouling Resistance in Membrane Distillation

**DOI:** 10.1021/acsami.3c12323

**Published:** 2023-11-14

**Authors:** Prexa Shah, Youmin Hou, Hans-Jürgen Butt, Michael Kappl

**Affiliations:** † Max Planck Institute for Polymer Research, Ackermannweg 10, 55128 Mainz, Germany; ‡ School of Power and Mechanical Engineering, Wuhan University, 430072 Wuhan, China

**Keywords:** desalination, membrane distillation, superhydrophobic, nanofilament coating, wetting, fouling

## Abstract

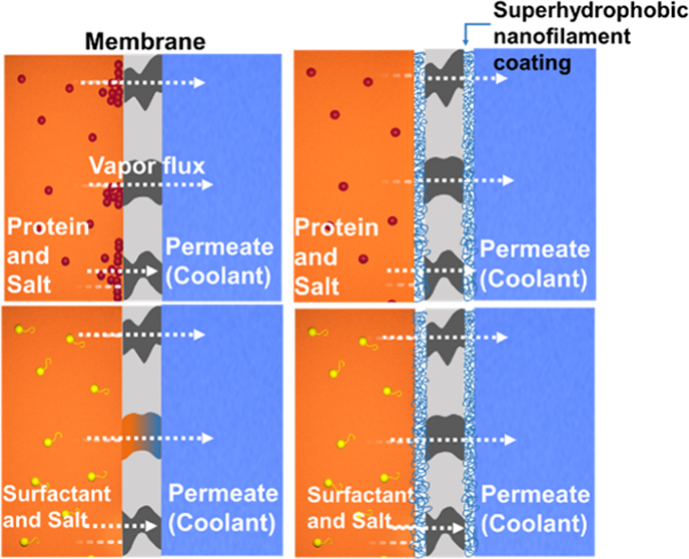

Membrane distillation (MD) is an important technique for brine desalination and wastewater treatment that may utilize waste or solar heat. To increase the distillation rate and minimize membrane wetting and fouling, we deposit a layer of polysiloxane nanofilaments on microporous membranes. In this way, composite membranes with multiscale pore sizes are created. The performance of these membranes in the air gap and direct contact membrane distillation was investigated in the presence of salt solutions, solutions containing bovine serum albumin, and solutions containing the surfactant sodium dodecyl sulfate. In comparison to conventional hydrophobic membranes, our multiscale porous membranes exhibit superior fouling resistance while attaining a higher distillation flux without using fluorinated compounds. This study demonstrates a viable method for optimizing MD processes for wastewater and saltwater treatment.

## 1 Introduction

Water shortage and the need for desalination and purification of wastewater have generated significant interest in membrane distillation (MD). In MD, the hot feed and cold distillate streams are separated with a porous, hydrophobic membrane. Because of the temperature difference, water evaporates at the membrane–feed interface and passes as vapor through the membrane pores, condensing as freshwater on the permeate side.^1,2^

MD has a strong rejection of nonvolatile compounds, a lower operating pressure than that of reverse osmosis, and a lower operating temperature than that of multistage flash distillation. It still requires a substantial amount of heat, so for normal desalination, reverse osmosis is often more efficient. For this reason, the strength of MD is for the treatment of high-salinity or highly concentrated wastewater. It can use low-grade heat sources such as waste heat from industries.^3−5^ To build an integrated separation system, the MD process can be paired with another separation process, such as ultrafiltration or reverse osmosis.^6,7^ Furthermore, MD has the capability of using alternative energy sources such as solar energy.^8,9^ Because of its compact footprint, MD has recently been recommended for off-grid applications.^10^

In addition to hydrophobicity, an ideal membrane for MD should be mechanically robust, have a high water vapor penetration flux, and have low thermal conductivity to reduce heat loss. Due to their low surface energy, the majority of membranes used in MD are composed of polyethylene (PE), polypropylene (PP), poly(tetrafluoroethylene) (PTFE), or poly(vinylidene fluoride) (PVDF).^11−14^

Just recently, implementation of MD has diversified and reached out to areas beyond desalination such as brine concentration, recovery of critical resources, and removal of toxic compounds from water.^15−19^ Such applications impose a major challenge in MD because of scaling and fouling. Both decrease the membrane permeability due to the accumulation of unwanted deposits on the membrane surface and inside the membrane pores. Contaminants such as proteins tend to attach to hydrophobic surfaces and also inside the pores because of the attractive hydrophobic interaction. Then, they block the membrane pores and reduce vapor diffusion.^20−24^ In addition to fouling, surface-active contaminants may reduce the surface tension of the feed solution and lead to wetting of the pores with a resulting breakthrough.^25−30^ A promising approach to reduce fouling is employing superhydrophobic membranes. Superhydrophobic membranes have superior antiwetting capabilities and many researchers have demonstrated that utilizing superhydrophobic membranes for MD reduces fouling. Superhydrophobic surfaces are known for their large water contact angles (>150°) and extremely low roll-off angles (<5°). The use of superhydrophobic surfaces is not restricted to membrane distillation but has a wide range of additional applications.^31−33^ The bulk of superhydrophobic membranes is made via fluorination.^34−38^ Fluorinated organic compounds are, however, environmentally detrimental and are no longer considered acceptable.

Our recent research suggests fluorine-free superhydrophobic coatings made by depositing a thin layer of silicone nanofilament network onto the surface of a microporous core membrane.^39^ In this prior research, the developed membranes demonstrated excellent distillation performance in comparison with previous studies for pure saltwater in the feed solution (for a detailed comparison, see Supporting Information Figure S2). Furthermore, these membranes were evaluated for long-term distillation, and they showed no drop in distillation flux, conveying no scaling, and salt rejection >99.9%, indicating no wetting. As a result, we focused our research on the fouling and wetting behavior of these extremely water-repellent nanofilament-coated membranes. Bovine serum albumin (BSA) was utilized as a model organic foulant for proteins to assess the antifouling performance of membranes. The antiwetting characteristics were tested with sodium dodecyl sulfate (SDS) solutions to investigate the impact of surfactants on membrane pore wetting. We demonstrate that our fluorine-free composite membranes have excellent antifouling and antiwetting properties, extending the range of MD applications.

## 2 Materials and Methods

### 2.1 Materials and Chemicals

As membranes, we used a flat sheet PE membrane with a nominal pore size of 0.2 μm and an average thickness of 110 μm from Lydall Performance Materials, a PTFE membrane with a nominal pore size of 0.2 μm and an average thickness of 150 μm from Donaldson Filtration Solutions, and hydrophilic poly(ether sulfone) (PES) membranes with nominal pore sizes of 1.2 and 8 μm and a thickness of 110–150 μm from Sterlitech Corporation. Bovine serum albumin (BSA) and sodium dodecyl sulfonate (SDS) were purchased from Sigma-Aldrich and MP Biomedicals Germany GmbH, respectively.

### 2.2 Fabrication of the Nanofilament (NF)-Coated Membrane

**Figure 1:**
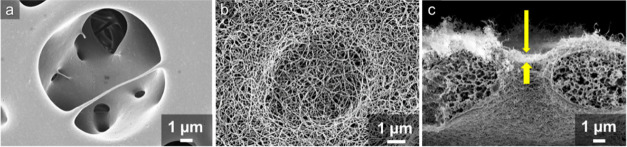
Scanning electron microscopy (SEM) images of the pristine PES-8 membrane (a), nanofilament-coated PES-8 membrane (b), and cross-section of nanofilament-coated PES-8 with hierarchical porous structures. Yellow arrows in (c) denote the nanoporous outer layer on top of microporous structures.

PES membranes were first activated via oxygen plasma (Diener Electronic Femto, 90 W for 2 min at a flow rate of 6 cm^3^/min) to create hydroxyl groups on the surface. Following plasma activation, the hydrophilic membranes were submerged in a 1:1 *n*-heptane/toluene mixture containing 0.017 M trichloromethylsilane and trace quantities of water (180 ppm). Under such conditions, trichloromethylsilane hydrolyzes and reacts with hydroxyl groups on the membrane surface, leading to the formation of a dense porous network of polysiloxane nanofilaments (Figure 1) (for details on the chemical reaction, see Supporting Information Figure S3).^40,41^ These nanofilaments (NFs) exhibit low surface energy due to their chemical nature. Their coiled and interwoven structure results in a local overhanging topography with an inward curvature. This combination of low surface energy and surface topography makes the NF coating superhydrophobic and capable of stabilizing an air cushion underneath the liquid–solid interface, thus maintaining the Cassie–Baxter wetting state.

As seen in Figure 1b, the NFs form a network structure that completely covers the top surface of the PES membrane, including the big pores. Even with a nominal pore diameter of 8 μm for a PES-8 membrane, the NFs can cover the pores on the top surface. Figure 1c illustrates a cross-section of the PES-8 membrane with the NF coating. We can clearly observe a thin (∼1 μm) layer of NFs created on top of the membrane. Growth of NFs within the inner porous structure does occur as well (Figure 1c), but the formed inner coating is sufficiently thin to avoid a significant change in the membrane porosity and pore size. As the maximum thickness of the NF layer inside the membrane is ∼500 nm, we can expect that the inner coating will not significantly hinder the vapor diffusion inside the membranes. In the following, the terms NF-PES-1.2 and NF-PES-8 refer to nanofilament-coated PES membranes with nominal pore diameters of 1.2 and 8 μm, respectively.

Energy-dispersive X-ray (EDX) analysis of the NF layer showed the presence of oxygen, silicon, and carbon, as expected for a polysiloxane material (see Supporting Information Figure S1).

### 2.3 MD Antiwetting and Antifouling Tests

A custom-made air gap (AGMD) and direct contact (DCMD) setup was established to test the antifouling and antiwetting performance of commercial and developed membranes (see Supporting Information Figure S1). These included an AGMD and DCMD module as well as feedwater and coolant circulation loops, a digital weight balance, a conductivity meter, and a data acquisition system (Figure S1a). The tested membrane was installed in an appropriate module. A cooling water bath circulator was employed in the coolant flow loop (F25-HE, Julabo). For calculating the distillation flow of the tested membranes, a digital balance (SPX 2202, Ohaus) consistently recorded the weight of the collected distilled water. The conductivity meter was used to monitor the conductivities of feed- and distilled water in order to calculate salt rejection during MD. Based on the weight and conductivity of the produced water, the distillation flux and salt rejection are computed using the equations given below^42^
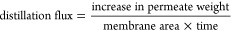
1
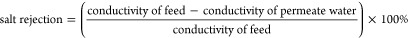
2

To monitor the liquid temperature at the inlet and exit of the feed flow channel and coolant flow channel, four Pt100 temperature probes (PM-1/10–1/8–6–0-P-3, Omega) were used. To continuously monitor the flow rate and pressure in the feed and coolant loops, two flow meters (FT110, Gems) and two pressure transducers (IPSLU-M12, RS-Pro) were placed in the pipelines. The MD testing setup’s sensors were all electrically coupled to a data-collecting system comprising two National Instruments (NI) analogue input modules (PCI 6251 and NI-9216). Throughout the MD tests, the measured data were sent to the computer, which could be watched in real time and saved using self-written LabView programs. Detailed information can be found in the Supporting Information (Figure S4).

To test the effect of fouling on MD, we used 1 g/L BSA and 35 g/L NaCl in the feed. The temperature of the inlet feed solution containing salt and BSA (*T*_f_) was 53 °C. This relatively low temperature was chosen based on the fact that above 53 °C, BSA starts to denature and coagulate. This coagulation leads to the formation of large aggregates that attach to the pipes of the distillation setup and result in the complete clogging of the whole circulation system. The temperature of the distillate inlet stream (*T*_c_) was 15 °C. The feed and distillate volume flows for AGMD were 1 and 2 L/min, respectively. For DCMD experiments, the feed and distillate volume flows were 1 and 0.3 L/min, respectively.

To test the effect of the surfactant on MD with NF-coated membranes, we used 2.8 and 56 mg/L SDS with 35 g/L NaCl in the feed solution (surface tensions: 55 and 35 mN/m, respectively) for AGMD. This concentration of SDS was chosen since the surfactant’s critical micelle concentration (CMC) in the presence of salt is 56 mg/L.^25^ The temperatures used were *T*_f_ = 65 and 80 °C and *T*_c_ = 15 °C. The feed and distillate volume flows were 1 and 2 L/min, respectively. In DCMD, membranes are more prone to wetting since they are in direct contact with water on both sides. Therefore, less severe wetting conditions were chosen. The DCMD tests were conducted with 28 mg/L SDS and 35 g/L NaCl (surface tension: 41 mN/m) with feed and distillate volume flows of 1 and 0.3 L/min, respectively. *T*_f_ was 60 °C, and *T*_c_ was 20 °C.

### 2.4 Membrane Characterization

EDX elemental analysis of the nanofilament coating was performed using an EDX system (EDAX Genesis XM4i) integrated into an FEI Nova 600 Nanolab FIB/SEM system. To avoid sample charging during SEM imaging, samples were coated with a 7 nm platinum layer (CCU-010 HV high-vacuum compact coating unit, Safematic GmbH).

Scanning electron microscopy (SEM Zeiss LEO 1530 Gemini) was used for examining the membrane surface morphology before and after exposure to BSA solutions. To avoid sample charging during SEM imaging, samples were coated with a 7 nm platinum layer by sputtering (CCU-010 HV high-vacuum compact coating unit, Safematic GmbH). PE, PTFE, and NF-coated PES membranes were immersed in a BSA solution of 0.5 g/L for 24 h. Then, the membranes were washed with Milli-Q water and dried under a nitrogen stream before SEM imaging.

To observe protein adsorption in situ, we used a confocal laser scanning microscope (TCS SP8 from Leica Microsystems GmbH, Germany, 20× objective, 0.50 numerical aperture). BSA was labeled with the fluorescent dye Nile Red. Nile Red, a fluorescent hydrophobic dye, provides for the efficient, sensitive, and wide staining of proteins.^43,44^ BSA has good solubility in water; however, Nile Red is sparingly soluble. To attach the dye to BSA, a pinch of Nile Red was added to 30 mL of aqueous BSA solution and stirred at 600 rpm overnight. The polarity of the environment in which Nile Red is dissolved affects its fluorescence. Nile Red is very fluorescent but only in a hydrophobic environment. In one study, the emission maximum was found to be changed from around 665 nm (red) in water to approximately 587 nm (green) in dioxane.^45,46^ Membranes to be tested were glued to an open-flow channel sticky glass slide. The inlet and outlet of the flow channel were connected to a peristaltic pump. This pump was used to control the continuous flow of BSA/Nile Red solution through the channel. Membrane surfaces facing the flow channel were observed with the confocal microscope, while the BSA solution continuously flowed along them. The fluorescence of Nile Red was detected by two separate channels in the red (688 nm) and green (561 nm) spectral ranges. Nile Red emits red when in a hydrophilic environment like water, whereas it emits green when in a hydrophobic environment like the membrane surfaces used for MD.

To characterize the wetting properties of membranes in the presence of the surfactant, we measured the advancing and receding contact angles of sessile drops using a goniometer (DataPhysics OCA35). The effect of SDS on surface tension depends on both salt and temperature.^47,48^ Since in the existing literature, the combined effect of salt and temperature has not been reported so far, we have done the corresponding measurements of surface tension for high salt concentration at elevated temperatures (Supporting Information Table S1). We prepared mixtures of 56 mg/L SDS and 35 g/L NaCl (surface tension: 35 mN/m). The presence of SDS on the membranes was detected using attenuated total reflectance Fourier transform infrared spectroscopy (Bruker Platinum ATR spectrometer).

## 3 Results and Discussion

### 3.1 Fouling Resistance of Superhydrophobic NF-PES Membranes

**Figure 2:**
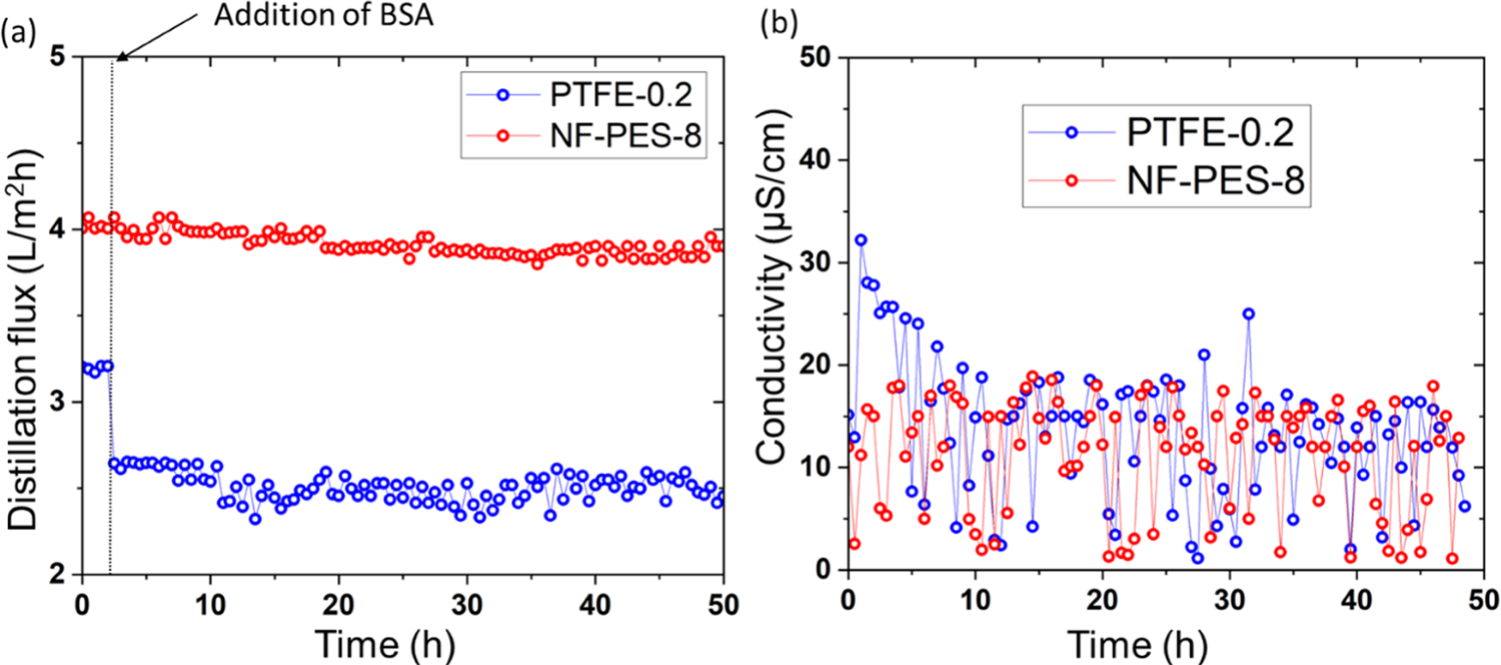
(a) AGMD distillation flux and (b) distillate conductivity as a function of time for original PTFE 0.2 and NF-PES-8 membranes (feed temperature *T*_f_ of 53 °C, distillate temperature *T*_c_ of 15 °C) with only 35 g/L salt and later with 1 g/L BSA and 35 g/L salt in the feed solution.

PTFE membranes are considered highly hydrophobic and having a good repellency against foulants. Therefore, commercial PTFE-0.2 membranes were chosen as a benchmark in MD tests. Figure 2 shows the influence of BSA on the distillation flux for PTFE 0.2 and NF-PES-8 membranes in the AGMD setup. Distillation was started with 35 g/L NaCl feed solution. NF-PES-8 membranes showed a distillation flux of ∼4 L/m^2^h, which is ∼25% higher than that of PTFE-0.2. When switching to a BSA-containing feed solution, the PTFE-0.2 membrane showed a sharp decline in the distillation flux from ∼3.3 to 2.6 L/m^2^h (∼23% decline) directly after restarting the distillation experiment with the new feed solution. The NF-PES-8 membrane was almost unaffected with only a ∼2% decline in flux over 48 h of AGMD experiments. The conductivity of the distillate obtained was always in the range of distilled water in both membranes.

**Figure 3:**
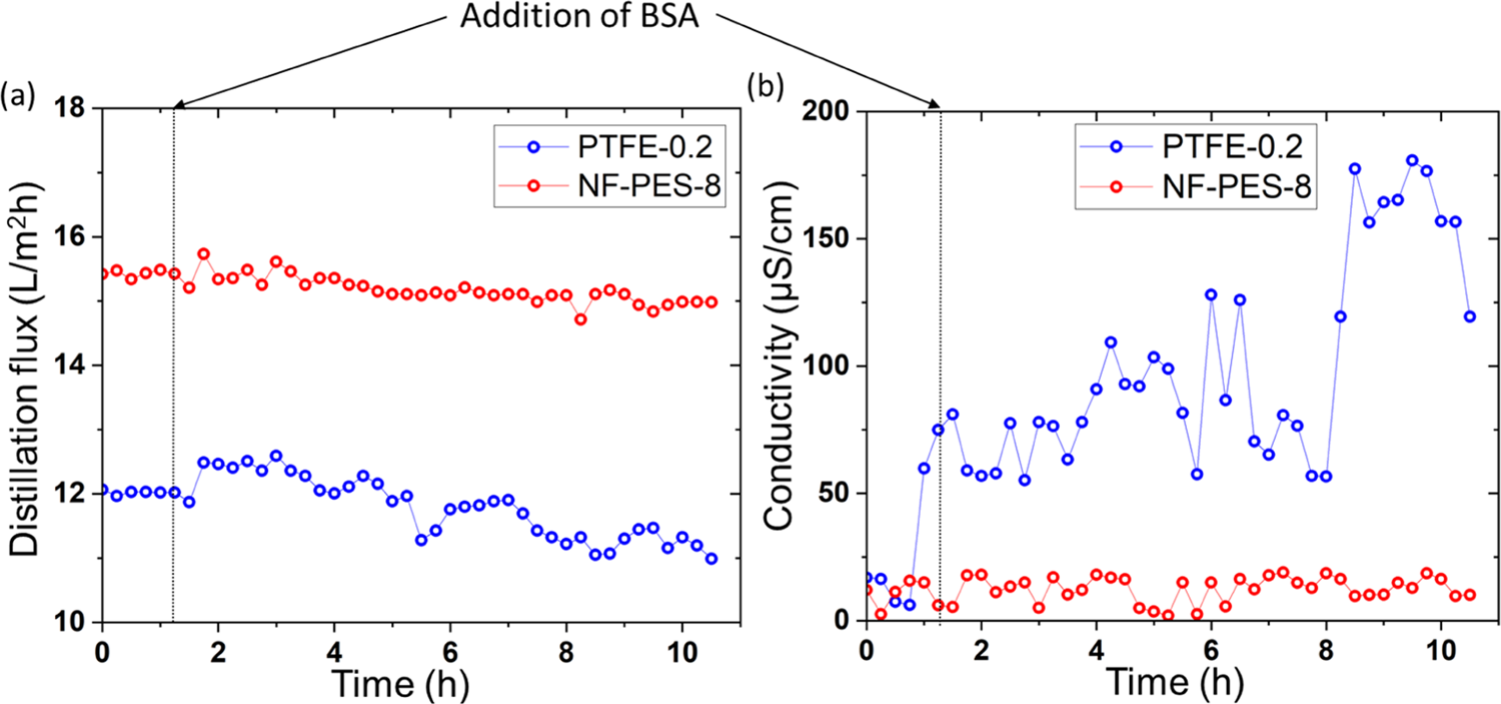
(a) DCMD distillation flux and (b) distillate conductivity as a function of time for original PTFE 0.2 and NF-PES-8 membranes (feed temperature *T*_f_ of 53 °C, distillate temperature *T*_c_ of 15 °C) with only 35 g/l salt and later with 1 g/L BSA and 35 g/L salt in the feed solution.

In DCMD experiments with only salt in the feed solution, the NF-PES 8 membrane showed a distillation flux of ∼15.5 L/m^2^h, which is ∼30% higher compared to that of PTFE-0.2. When switching to the BSA-containing solution, the flux through PTFE-0.2 decreased from ∼12 to 11 L/m^2^h (∼8% decline) over a period of 9 h with partial wetting as the conductivity of the distillate increased to 180 μS/cm (Figure 3). In contrast, the NF-PES-8 membrane showed a stable flux for 9 h with distillate conductivity always in the range of distilled water (salt rejection >99.9%). This proves that the superhydrophobic nanoporous structures increase the fouling resistance.

Due to the increased heat transfer across the membrane in DCMD, it was difficult to maintain a constant permeate temperature of 15 °C for a longer period of time in our setup that was originally designed for AGMD operation. Therefore, DCMD data were collected for 10 h only.

**Figure 4:**
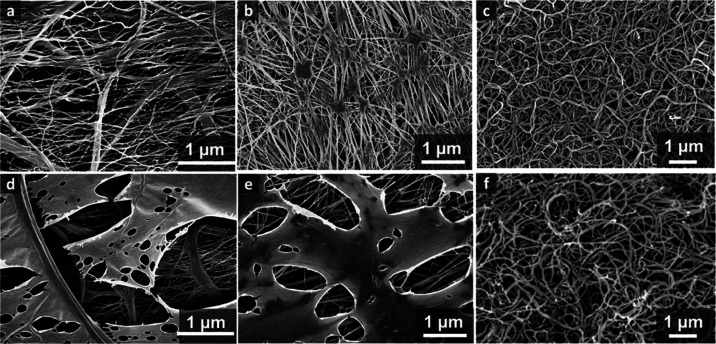
SEM images of (a–c) virgin PE, PTFE, and NF-coated PES membranes and (d–f) PE-, PTFE-, and NF-coated PES membranes after 24 h of immersion in 500 mg/L BSA solution.

To test if BSA adsorbs to membranes, all the membranes were immersed in 500 mg/L BSA salt solution for 24 h. For NF-coated PES membranes, we could not identify any adsorbed protein by SEM (Figure 4f). In contrast, layers of adsorbed BSA cover PE and PTFE membrane surfaces (Figure 4d,e). Since we never observed such layers for the immersion test in pure salt solution (even after 1 week of immersion), we can be sure that the observed layers are due to BSA adsorption. Thus, NF-coated PES membranes are less prone to fouling by proteins than commercial hydrophobic membranes, which have also been reported by other studies.^20,49,50^

To monitor BSA adsorption in real time, we mounted the membrane inside a flow channel and exposed it to a cross-flow of solution parallel to the membrane surface while imaging the membrane surface by confocal microscopy. The solution contained Nile Red-stained BSA, and the dye was excited with a 458 nm argon laser. The objective was focused near the membrane/water interface. Then, dyed BSA was allowed to flow along the membrane surface. The measurements were performed within a central 1024 × 256-pixel frame. Total fluorescence intensity was measured for 1 h (3600 s) with a rate of 0.357 frames/sec. The result was saved as a video data file and then analyzed using Fiji software, giving the plot of the fluorescence intensity over time for the respective membranes.

**Figure 5:**
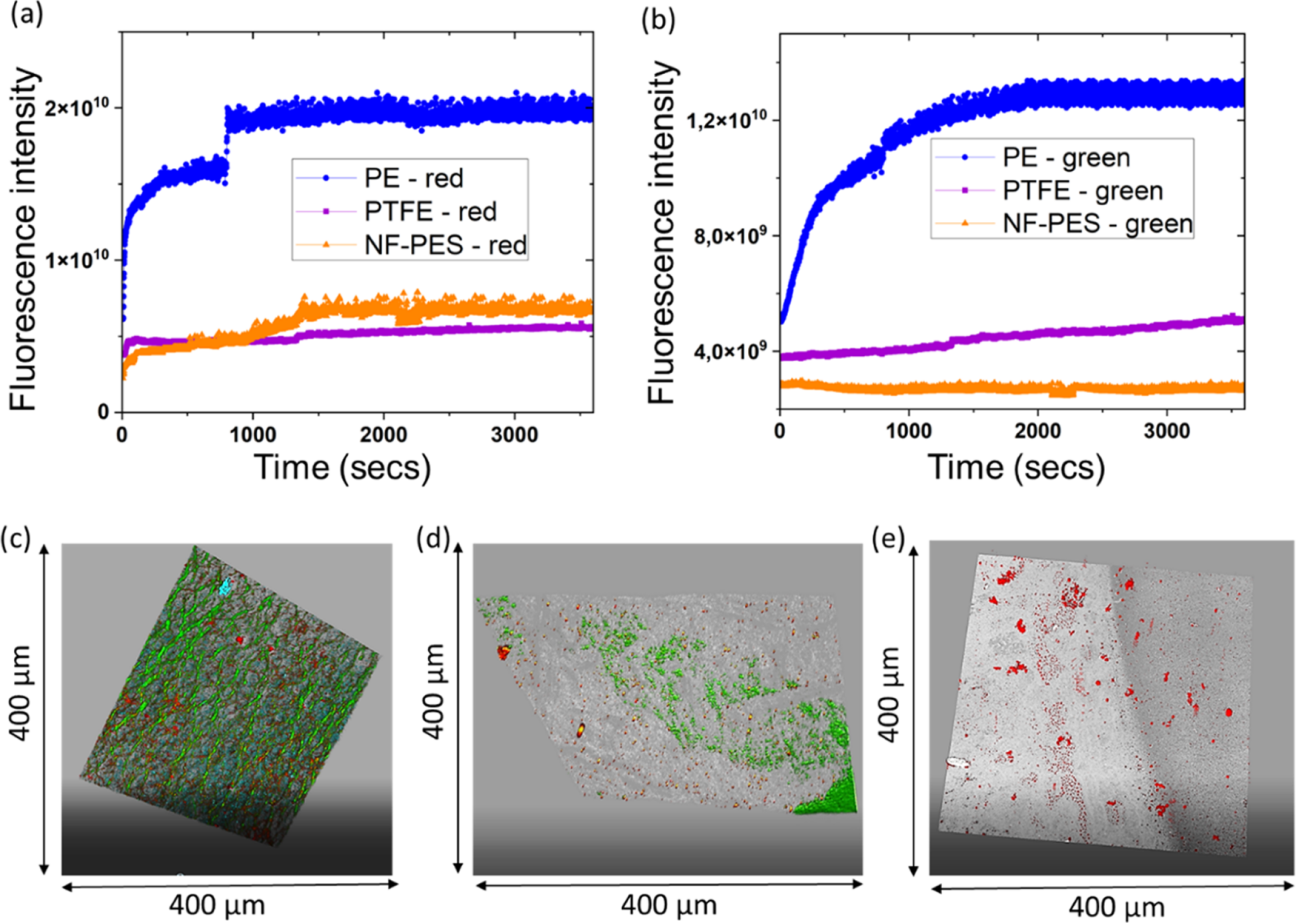
Confocal microscopy data. (a) Fluorescence intensity as a function of time for the red spectral range, indicative of BSA adsorbed in a hydrophilic environment. (b) Fluorescence intensity as a function of time for the green spectral range, indicative of BSA adsorption in a hydrophobic environment. Confocal images for (c) PE, (d) PTFE, and (e) NF-PES membranes (overlay of both green and red fluorescence).

PE membranes show stronger adsorption of BSA than NF-coated and PTFE membranes. Figure 5a gives the increase in the intensity of red fluorescence over time for PE, PTFE, and NF-coated membranes. Considering that the red fluorescence intensity is equivalent to BSA that is still mostly in contact with water, this would correspond to a weak adsorption to the membrane. It is observed that PE membranes show about 3 times more such weak BSA adsorption than NF-coated membranes. PTFE and NF-coated membranes exhibit a similar lower amount of adsorption.

Figure 5b gives the increase in the intensity of green fluorescence over time for PE, PTFE, and NF-coated membranes. Considering that the emitted green fluorescence intensity is equivalent to BSA no longer in contact with water, it should resemble strong adsorption on the membrane surface. PE does show approximately 3 times more strong BSA adsorption than that of nanofilament-coated PES membranes. Also, over time, PTFE membranes show roughly 2 times more such strong BSA adsorption than that of NF-coated PES membranes with an increasing trend. In the case of NF-coated PES, membranes exhibit very low and stable adsorption during 1 h of BSA solution flow.

Finally, from the confocal images in Figure 5c, it is observed that PE membranes show complete coverage with a green signal, confirming that protein BSA is adsorbed strongly and homogeneously on the membrane surface. PTFE membranes show BSA adsorbed on some parts of the membrane but not completely covered (Figure 5d). However, NF-coated PES membranes do not show any green signal, confirming very low and weak adsorption of BSA on the membrane surface (Figure 5e).

### 3.2 Wetting Resistance of NF-Coated Superhydrophobic Membranes in the Presence of a Low-Surface Tension Liquid

**Figure 6:**
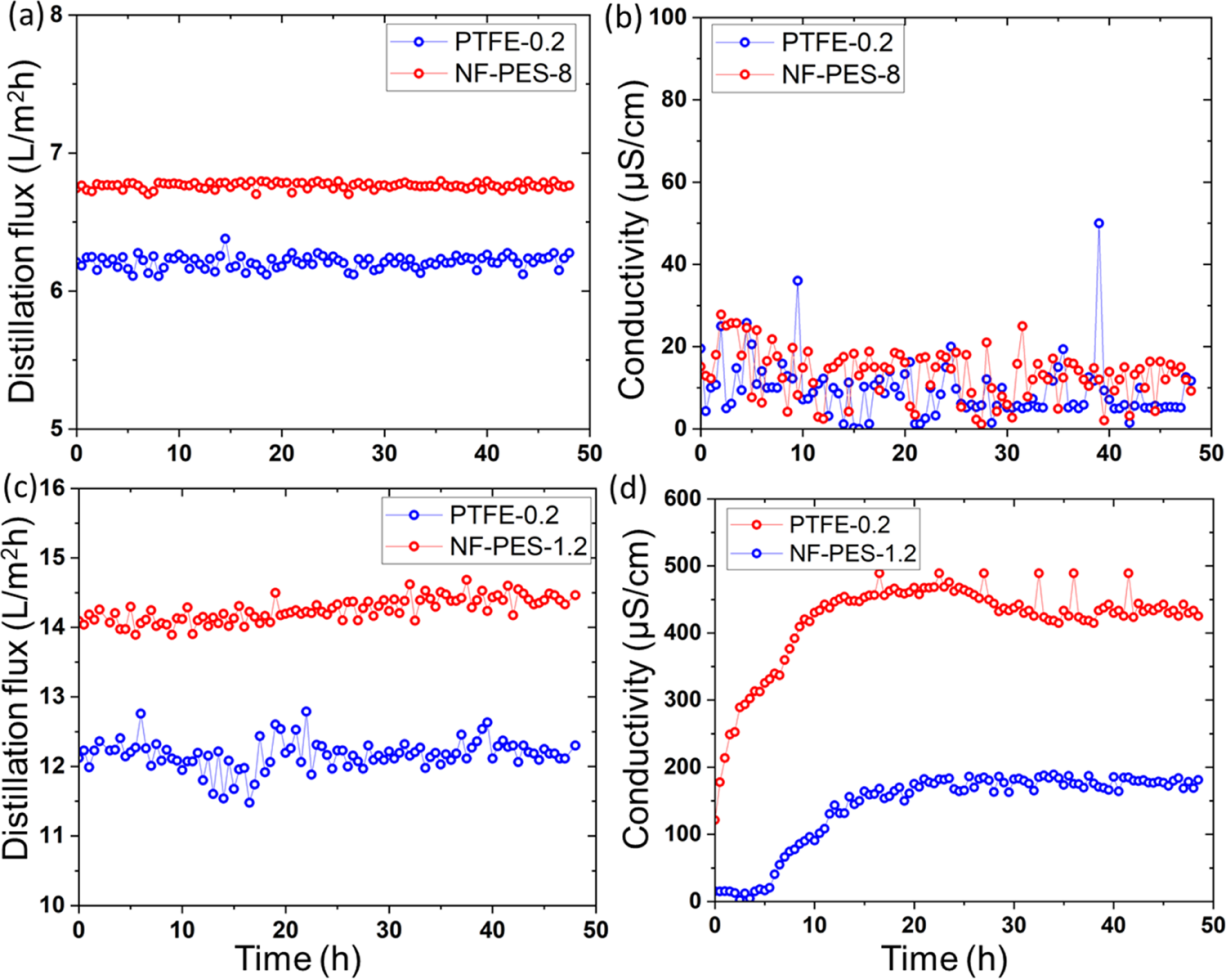
AGMD distillation flux and distillate conductivity over time (a, b) for original PTFE 0.2 and NF-PES-8 membranes with 2.8 mg/L SDS + 35 g/L salt (surface tension: 55 mN/m) at a *T*_f_ of 65 °C and *T*_c_ of 15 °C showing a stable flux for 48 h with no wetting (c, d) for original PTFE 0.2 and NF-PES-1.2 membranes with 5.6 mg/L SDS + 35 g/L salt (surface tension: 35 mN/m) at a *T*_f_ of 80 °C and *T*_c_ of 20 °C showing a stable flux for 48 h with partial wetting in both cases.

For investigating the antiwetting properties of the membranes during AGMD in the presence of surfactants, we used 2.8 mg/L SDS with 35 g/L NaCl in the feed solution (surface tension: 55 mN/m). The temperatures used were T_f_: 65 °C and *T*_c_: 15 °C. The feed and distillate volume flows were 1 and 2 L/min, respectively. It was observed that NF-PES-8 membranes exhibited a distillation flux of ∼6.7 L/m^2^h, which is ∼7% higher than that of PTFE-0.2 with a stable water flux for 48 h and distillate conductivity in the order of the distilled water range in both cases (Figure 6a).

After achieving successful results in the first trial, we also conducted AGMD experiments with a much higher concentration of 2.8 mg/L SDS with 35 g/L NaCl (surface tension: ∼35 mN/m). In the presence of a high concentration of SDS, the PTFE-0.2 membrane showed partial wetting. Distillate conductivity increased to ∼100 μS/cm at the beginning of the experiment and continued to increase to ∼500 μS/cm in 48 h using a feed temperature of *T*_f_ = 80 °C and a distillate temperature of *T*_c_ = 20 °C. The NF-coated PES-1.2 membrane was also tested at the same temperatures, where we observed a distillation flux of ∼14 L/m^2^h, which is ∼17% higher compared to that of PTFE-0.2. Under these harsher conditions, the NF-PES-1.2 membrane also exhibited a slight decrease in wetting resistance; the distillate conductivity increased to ∼200 μS/cm within 48 h (Figure 6b). Thus, in AGMD, the nanofilament-coated membranes outperform commercial membranes.

**Figure 7:**
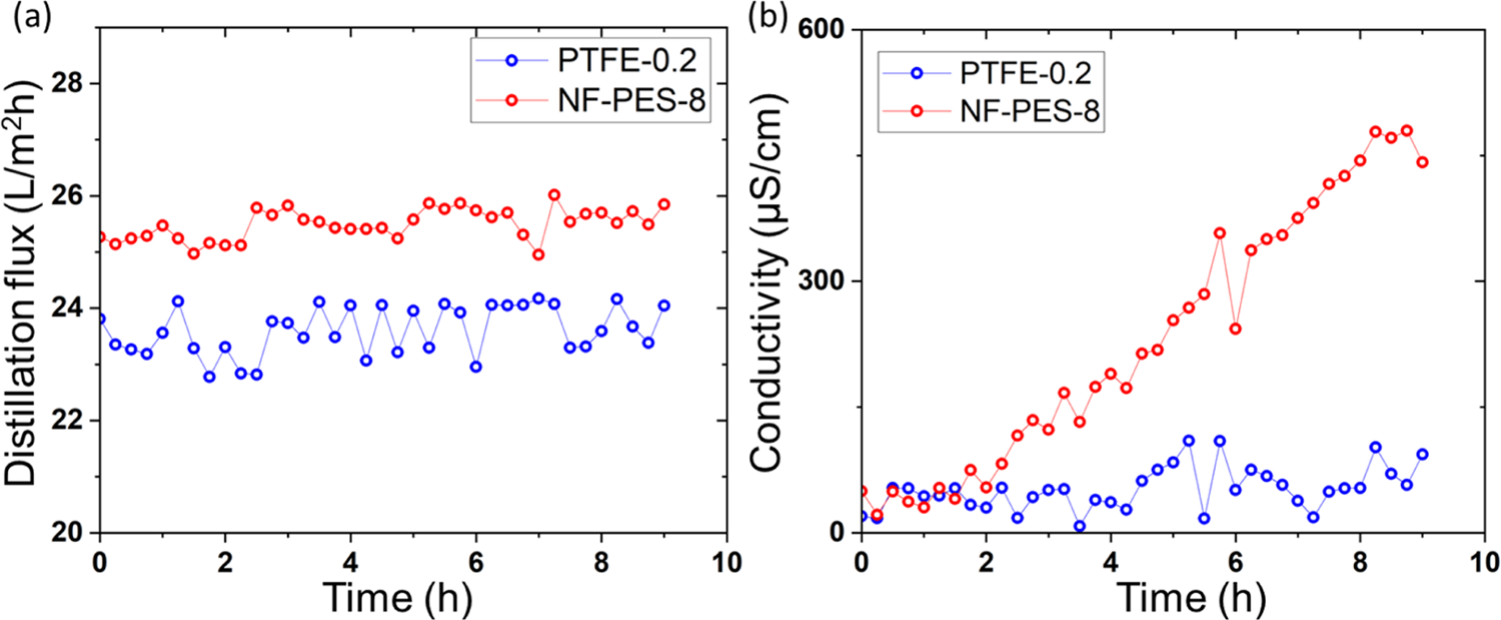
(a) DCMD distillation flux and (b) distillate conductivity over time for original PTFE 0.2 and NF-PES-8 membranes with 28 mg/L SDS + 35 g/L salt (surface tension: 41 mN/m) at a *T*_f_ of 60 °C and *T*_c_ of 20 °C indicating partial wetting for PTFE-0.2 membranes and a progressive rise in wetting for NF-PES-8 membranes.

DCMD was used to evaluate the membrane antiwetting performance. It should be noted that because the membrane is in contact directly with water on both sides in a DCMD setup, hydrophilic membranes that were rendered superhydrophobic may have a stronger tendency to get wetted for the low-surface tension feed. DCMD tests using SDS 28 and 35 g/L salt were conducted with PTFE-0.2 and NF-PES-8 membranes. As shown in Figure 7, the PTFE membrane showed minor wetting as the distillate conductivity increased to ∼150 μS/cm in 9 h. The NF-coated PES membranes showed a distillation flux of ∼25.1 L/m^2^h, which is ∼6% higher than that of PTFE-0.2, However, gradual wetting occurred after 2 h of testing and the distillate conductivity kept increasing continuously during the 9 h test.

**Figure 8:**
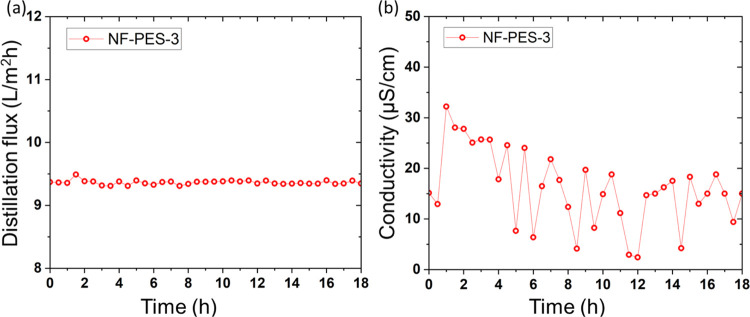
(a) DCMD distillation flux and (b) distillate conductivity over time for the original NF-PES-3 membrane with 28 mg/L SDS + 35 g/L salt (surface tension: 41 mN/m) at a *T*_f_ of 60 °C and *T*_c_ of 20 °C.

Our NF coating is quite thin and had been optimized for high flux, as shown in Figure 1c. However, this thin NF coating could not provide sufficient wetting resistance in the case of DCMD with a low-surface tension liquid (Figure 7b). By increasing the reaction time for NF growth, we can prepare thicker and denser layers. Using such a thicker coating for DCMD with a low-surface tension liquid, the distillation flux was reduced, but the distillate conductivity always lies in the distilled water range (Figure 8). This demonstrates that we can tune the properties to either maximize the distillation flux for high-surface tension liquids or optimize the wetting resistance for use in combination with low-surface tension liquids.

**Figure 9:**
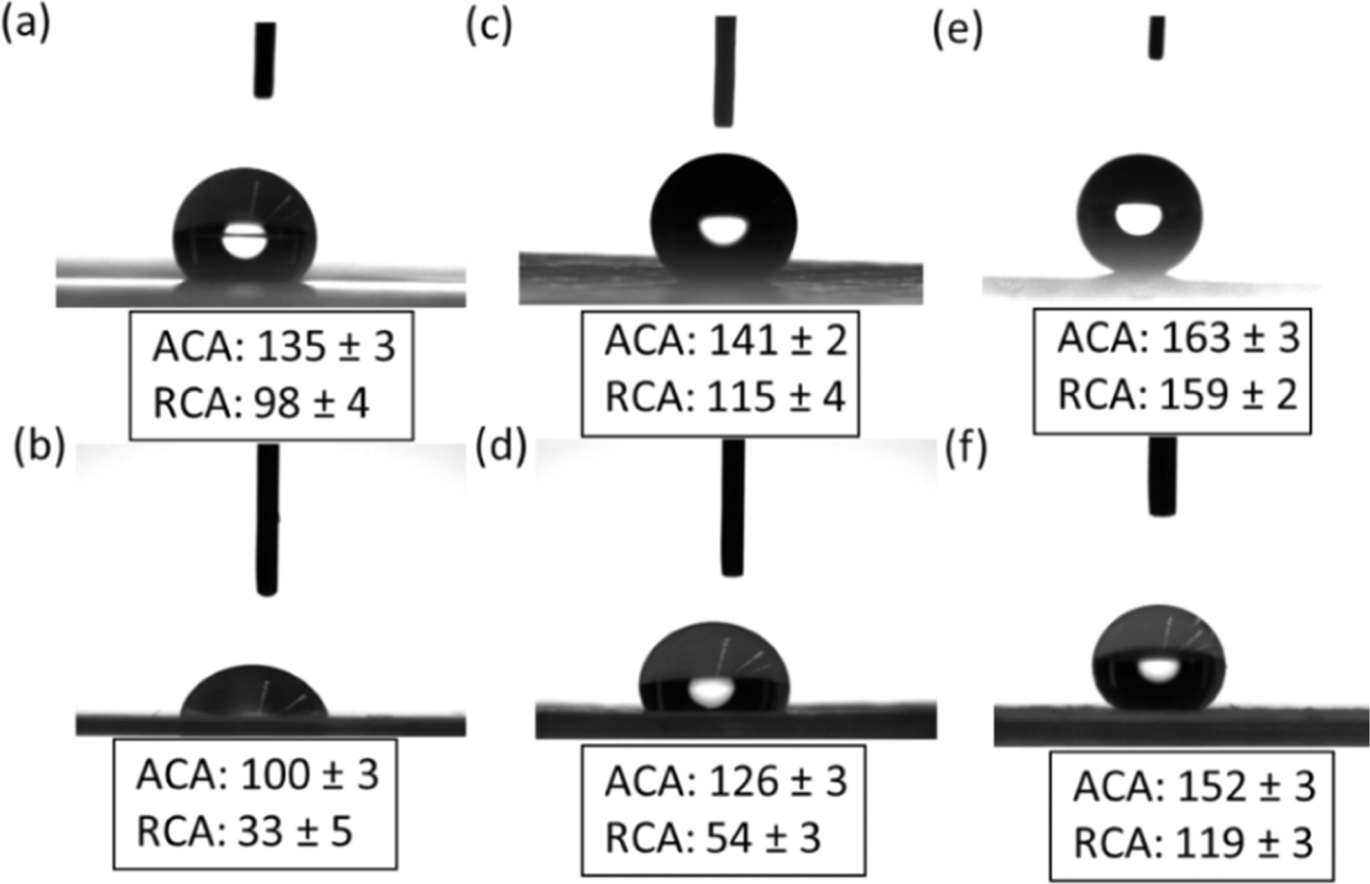
Advancing and receding contact angles (ACA and RCA) for pure water (surface tension of 72 mN/m): (a) PE, (c) PTFE, and (e) NF-PES membranes. ACA and RCA for 56 mg/L SDS + 35 g/L NaCl (surface tension of 35 mN/m): (b) PE, (d) PTFE, and (f) NF-PES membranes.

To characterize the wetting properties of the membranes in the presence of the surfactant, the advancing and receding contact angles were measured with either a 35 g/L NaCl solution or a solution containing 56 mg/L SDS and 35 g/L NaCl. In the case of the PE membrane, the presence of the surfactant led to a decrease of RCA from ∼98 to ∼33°, whereas CAH increases from ∼37 to ∼77°. CAH was calculated as the difference between advancing and receding contact angles. This observation indicates a considerable loss of liquid repellency (Figure 9a,d). Similarly, for commercial PTFE membranes, the RCA decreased from ∼115 to ∼54° and CAH increased from ∼25 to ∼67° (Figure 9b,e). The NF-coated PES membranes showed a much lower decrease in contact angles with an SCA of ∼147° and CAH of ∼31°. Hence, NF-coated PES membranes showed superior liquid repellency even with the SDS plus salt mixture having a surface tension as low as ∼35 mN/m (Figure 9c,f). Similar trends were observed for mixtures of water and ethanol, where liquid repellency was lost for PE and PTFE membranes but was still maintained for NF-coated membranes (Supporting Information Figure S6).

**Figure 10:**
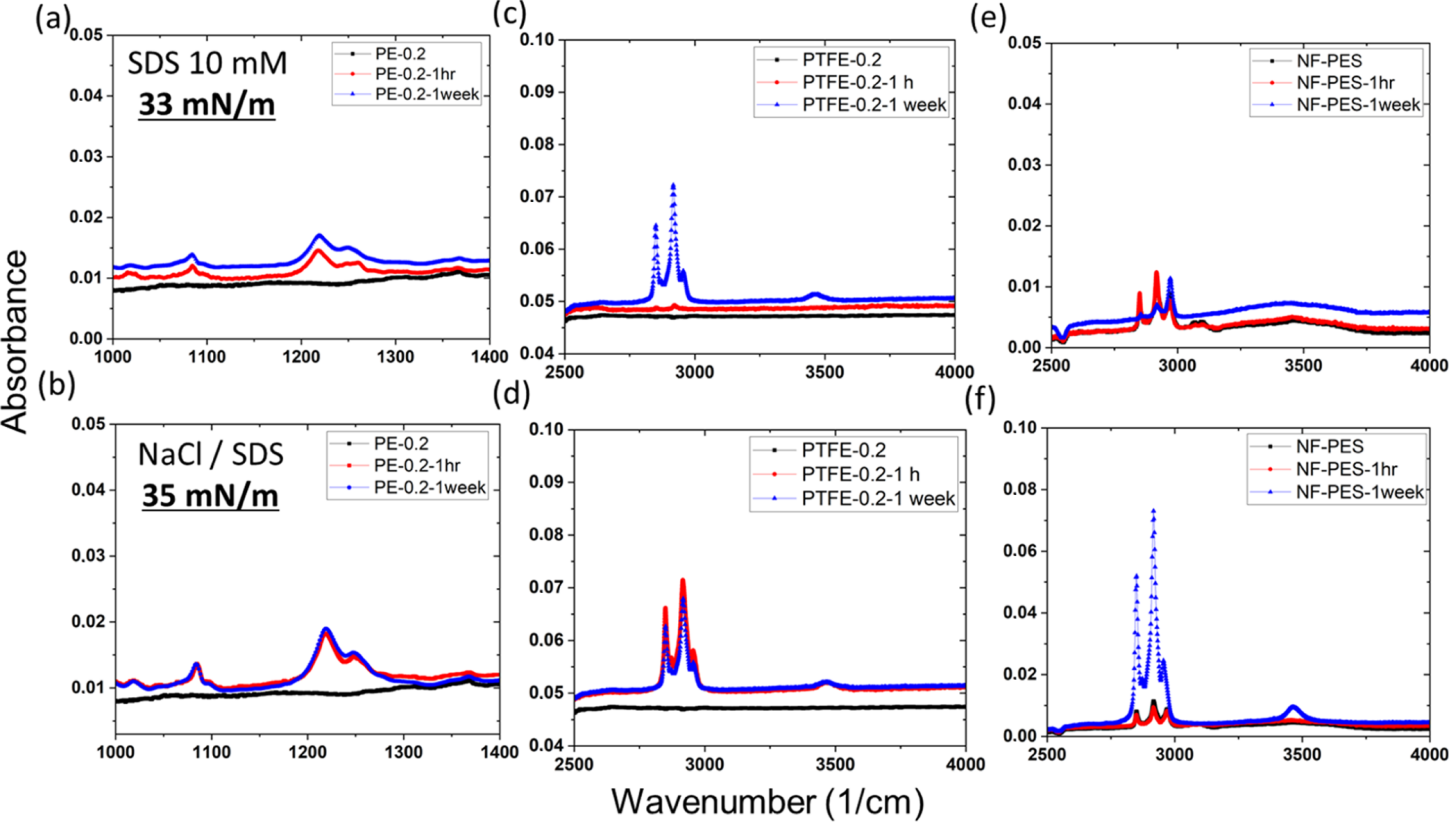
FTIR spectra for PE, PTFE, and NF-coated PES membranes for original and after SDS immersion in (a, c, e) 2.8 g/L SDS and (b, d, f) 56 mg/L SDS + 35 g/L salt for 1 h and 1 week of immersion.

To verify how quickly SDS, a low-surface tension mixture, gets adsorbed on MD membranes, PE, PTFE, and NF-coated PES membranes were immersed in SDS solutions with a concentration of 2.8 g/L (surface tension: 33 mN/m) and a mixture of SDS-56 mg/L + NaCl-35 g/L (surface tension: 35 mN/m) for 1 h and 1 week. To detect the presence of SDS on the membranes, a Fourier transform infrared spectrometer (FTIR) was used afterward. SDS has a distinctive peak at 1080 and 1216 cm^–1^ for S=O and S–O bonds, respectively, also for CH_2_ symmetric and asymmetric stretching at 2851 and 2926 cm^–1^, respectively.^51^^52^ Because it was unable to distinguish the difference in peaks from the membrane or SDS in PE membranes, the presence of SDS was detected by S–O and S=O bonds. Figure 10 shows the immediate adsorption of SDS on PE and PTFE membranes. On the contrary, NF-coated PES membranes did not show any distinct peaks except after 1 week of immersion in the mixture of salt and SDS, where the peak for CH_2_ symmetric stretching with higher intensity was observed (Figure 10e,f).

## 4 Summary and Outlook

The fouling and wetting resistances of our newly designed fluorine-free superhydrophobic membranes were investigated by using the protein BSA and the surfactant SDS. All of our membranes were shown to have fouling resistance that exceeded the antifouling capabilities of standard PTFE membranes. This was demonstrated using MD tests, SEM images, and confocal microscopy, where a layer of BSA was formed on the PTFE membrane surface, resulting in a 23% decrease in AGMD flux and an 8% decrease in DCMD flux. There is no such protein layer formation observed for NF-coated PES membranes, and the distillation flux is stable over time. The wetting resistance of all NF-coated membranes was found to be at least on par and in most cases exceeding the antiwetting properties of commercial PTFE membranes from MD tests, FTIR data, and contact angle measurements. Our NF-coated membranes showed almost 17% higher water distillate flux in the presence of SDS, while having at least the same wetting resistance as the PTFE-0.2 membrane in AGMD. A thin NF-coated PES membrane reaches its limit in DCMD with a low-surface tension liquid and starts to show wetting in this configuration. Although a thick NF-coated PES membrane compromises distillation flux, on the contrary, its salt rejection is >99 9%, making it a viable alternative to PTFE or other fluorinated membranes. This fluorine-free superhydrophobic NF coating technique is simple, readily reproducible, stable, and cost-effective and does not require any sophisticated equipment. This work will not only address the basic difficulties that have persisted in the MD process but also open the way for the use of advanced hierarchical porous membranes in a larger range of water treatment applications.

## Supporting Information Available

EDX data showing elemental analysis for surface elemental composition of nanofilament-coated PES membranes, comparison of membrane distillation performance with current state of the art membranes from literature, reaction mechanism for the formation of nanofilaments on the surface, measured surface tension values for SDS and NaCl solution, details of MD setup, SEM images of BSA adsorption after MD operation, and wetting properties for low surface tension liquids (ethanol mixtures) (PDF)
