# Supplementary material for: Nanofilament-Coated Superhydrophobic Membranes Show Enhanced Flux and Fouling Resistance in Membrane Distillation
Source: ACS Appl Mater Interfaces. 2023 Nov 14;15(47):55119–28. doi: 10.1021/acsami.3c12323 (PMC10694809; doi:10.1021/acsami.3c12323)
Supplement: Supplementary file 1 — am3c12323_si_001.pdf [file am3c12323_si_001.pdf]

Supporting Information

Hierarchical superhydrophobic composite membrane for enhanced distillation with excellent fouling resistance

Prexa Shah[1], Youmin Hou[2], Michael Kappl[1*], Hans-Jürgen Butt[1]

1. Max Planck Institute for Polymer Research, Ackermannweg 10, 55128 Mainz, Germany;

2. School of Power and Mechanical Engineering, Wuhan University, 430072, Wuhan, China

*Correspondence: kappl@mpip-mainz.mpg.de

Surface composition of nanofilament-coated membranes

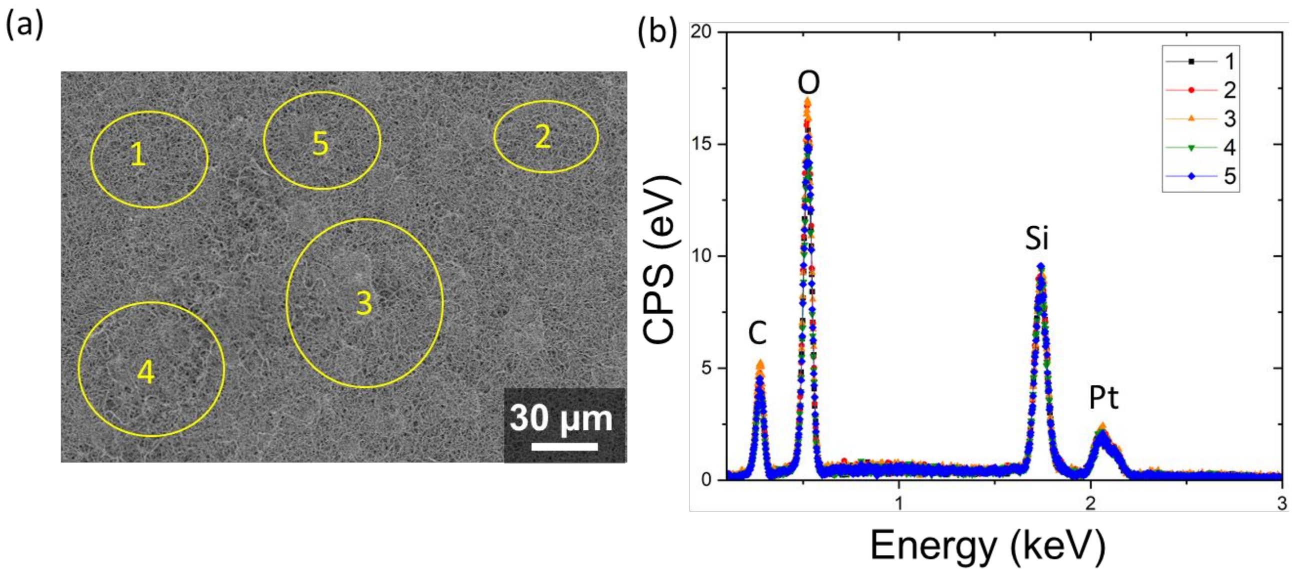

Figure S 1: EDX data showing elemental analysis for surface elemental composition of nanofilament-coated PES membranes a) SEM image of nanofilamtent coating with 5 different spots analysed by EDX b) EDX spectra of the five spots marked in a).

Figure S1 depicts an EDX analysis of nanofilament-coated membranes along with the corresponding SEM image. The presence of platinum on the surface is explained by the fact that the samples were first sputter-coated with a platinum layer. Other components included are polysiloxane nanofilament elements found on the membrane surface.

Comparison of membrane performance with previous studies

We compared the mass flux coefficient of the NF-PES membrane with earlier research of AGMD and DCMD to clearly describe membrane performance for water desalination, as shown in Figure S2. The mass flux coefficient was defined in this case as the ratio of distillation

flux to vapor pressure differential across the membrane. We compared the salt rejection of the membranes in AGMD tests and the LEP of the membranes in DCMD tests due to a lack of documented data in the literature. As shown in Figure S 2 a, the NF-PES membrane achieves top-tier performance in both mass flux coefficient and salt rejection, which were not obtained concurrently in prior AGMD test results.

As demonstrated in Figure S 2 b, the NF-PES membrane efficiently boosts the mass flux coefficient of the DCMD without compromising on the LEP. Furthermore, the high LEP ensures that the NF-PES membranes can withstand large hydraulic pressures, signifying a great potential for wastewater treatment.

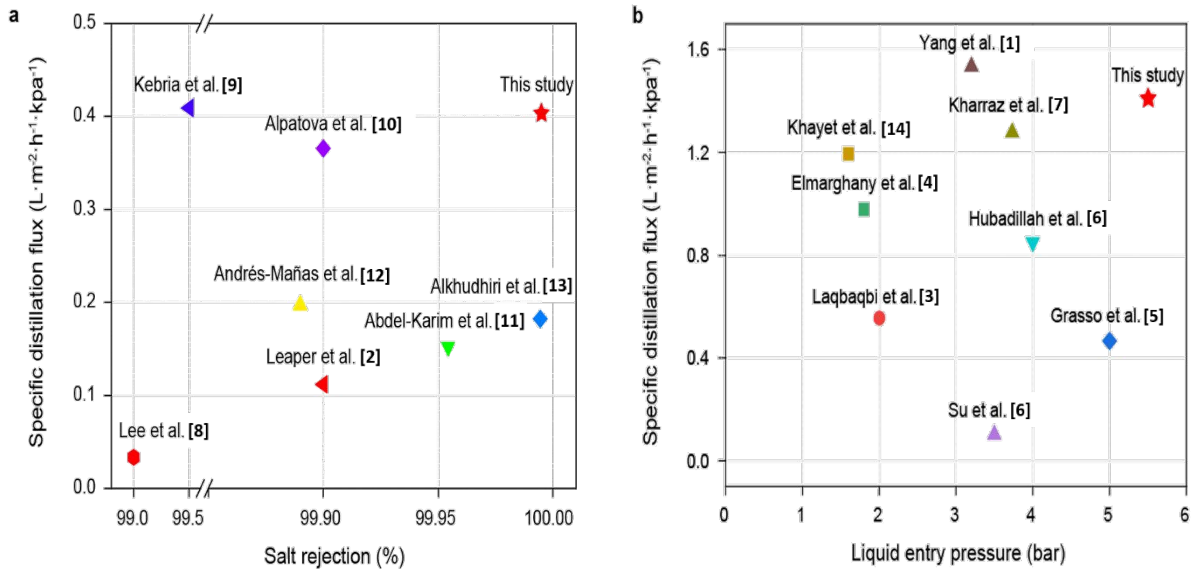

Figure S 2: Comparison of the (a) AGMD and (b) DCMD performances of the nanofilament-coated membrane with various previous reports [1-14]. For the AGMD test, the NF-PES membrane can simultaneously achieve high specific distillation flux and high salt rejection. Similarly, the NF-PES membranes effectively enhanced the distillation flux in the DCMD test without compromising the liquid entry pressure (LEP). Figure taken from [16], conforming to CC BY license.

Fabrication of nanofilament (NF) coated membrane

- surface activation to generate –OH groups. Surfaces need to have –OH groups as grafting points. This is achieved by oxygen plasma treatment.
- hydrolysis of trichloromethyl silane (TCMS) by trace amounts of water (~180 ppm) present in the organic solvent (toluene or n-hexane).
- hydrolysed TCMS reacts with –OH groups at the surface to form polysiloxanes.
- it is assumed that nanofilament growth is due to localized, diffusion-limited water adsorption at the surface reaction sites, leading to a 1D growth originating from these nanoscopic water reservoirs. But the exact molecular picture is still unclear.

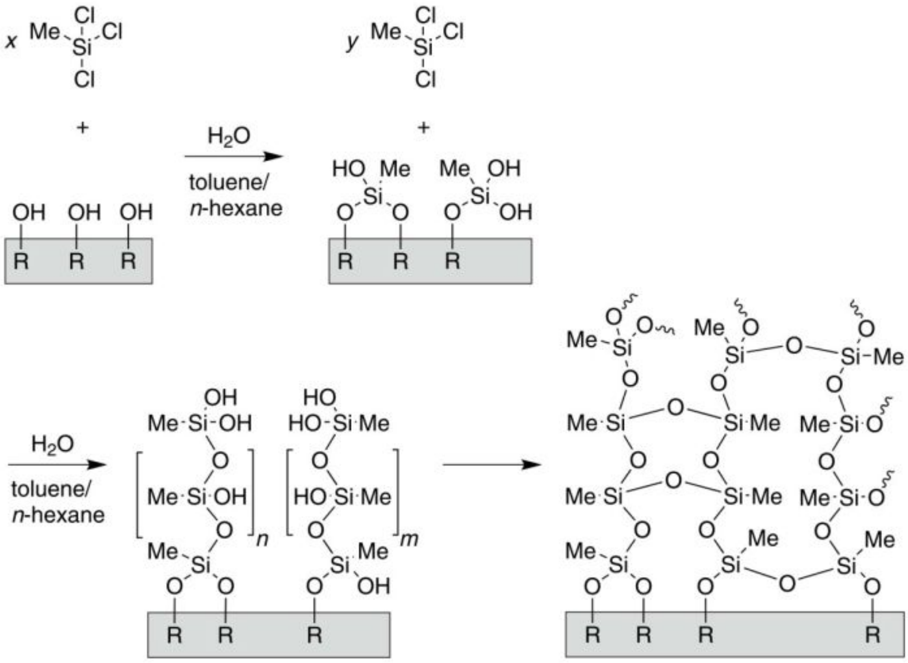

Figure S 3: Reaction mechanism for the formation of nanofilaments on the surface.

Surface tension of SDS / salt solutions

When salt is added to the SDS solution, there is a drastic drop in the surface tension of the solution (Table S 1). For characterizing the wetting properties, we used 0.1 mM and 0.2 mM of SDS with 0.59 M NaCl.

Table S 1: Measured surface tension values for SDS and NaCl solution

SDS (mM)	NaCl (M)	Temperature (^{o}C)	Surface tension (mN/m)
0.1	0	25	69 ± 2
	0.59	25	41 ± 1
	0.59	75	38 ± 1
0.2	0	25	55 ± 2
	0.59	25	36 ± 1
	0.59	75	35 ± 1

MD setup

A custom-made air gap membrane distillation (AGMD) setup was established to test the anti-fouling performance of commercial and developed membranes (Figure S 4). The membrane to be tested is placed between the feed flow channel and the condensing surface. Feed saline water is heated to the required temperature and pumped to the AGMD module. After the water vapor diffuses through the membrane, it is condensed on the condensing surface. The weight and conductivity of water produced are continuously measured using the weight balance and conductivity meter (Figure S 4 b). The flow channel on the inlet side had a width and length of of 7 cm. Flow rate was 1 L/min, ensuring laminar flow along the membrane. The module size was chosen to be sufficient to exclude significant influence of the entrance effect on the distillation performance [15].

All the AGMD experiments were carried out over 48 hours to test the membrane‘s durability for long-term operation.

Membranes were also investigated for anti-fouling performance in the direct contact membrane distillation (DCMD) module. The DCMD setup was built by removing the gap spacer and condensing surface used for the AGMD module. In this way, the membrane would be in direct contact with the feed and permeate side.

(a)

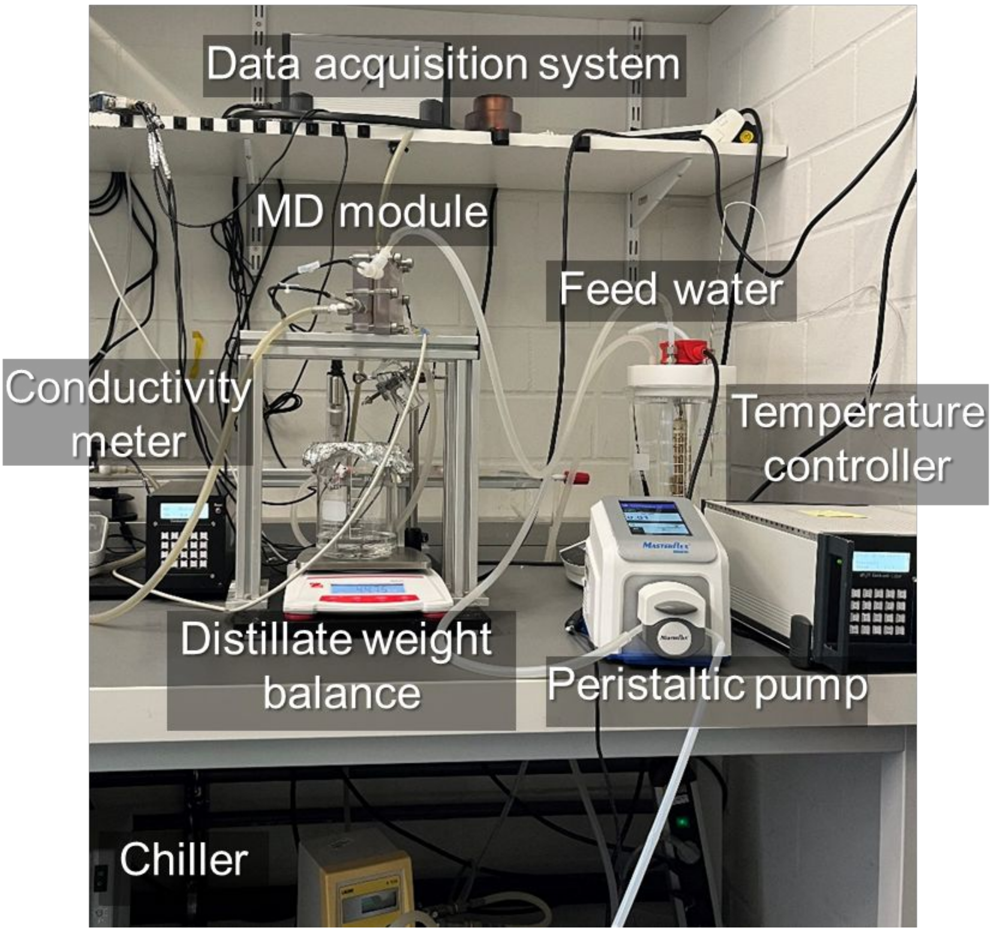

(b)

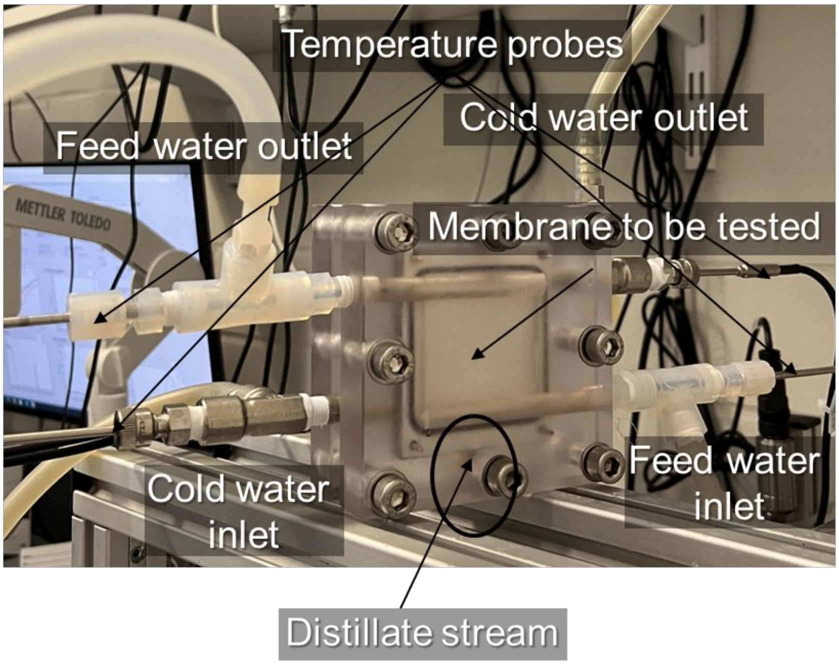

Figure S 4: (a) Image of AGMD lab testing setup (b) AGMD module

Adsorption of protein BSA on membrane

To check the adsorption of BSA to the membranes during membrane distillation, which could be indicative of possible biofilm formation, we did SEM imaging for the samples after the AGMD operation. Membranes were taken out of the distillation setup, shortly rinsed with distilled water, and then dried in the air before transferring them to the SEM. It was observed that for PTFE-0.2, a layer of BSA forms on the membrane surface covering some areas completely and having cracks in some areas that expose the bare membrane structure (Figure S 5 b).

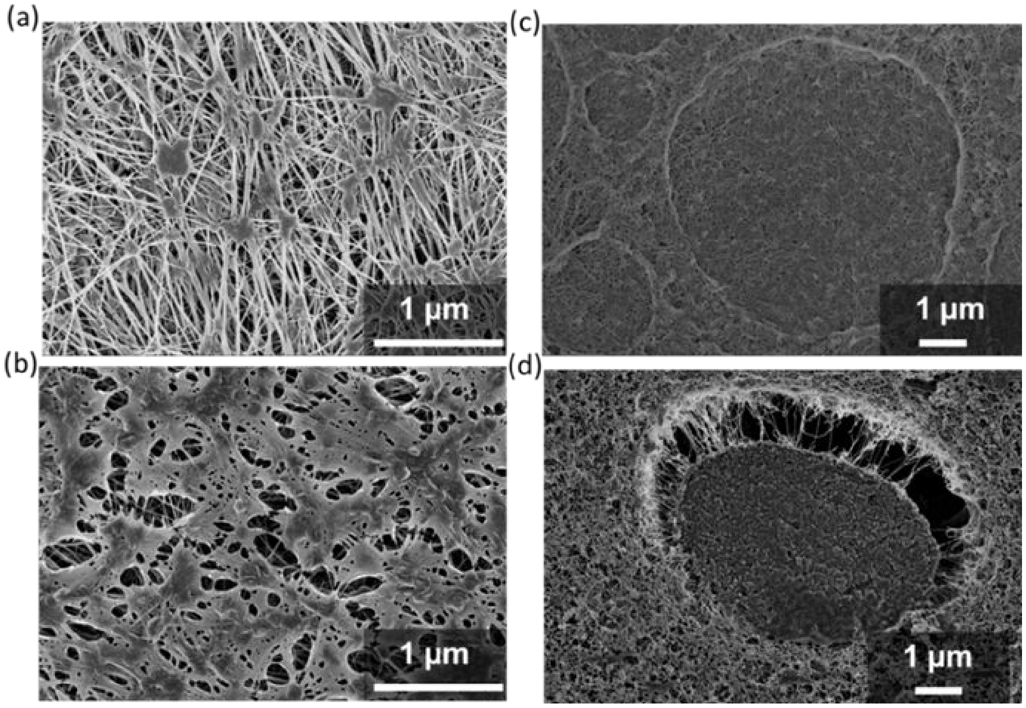

Figure S 5: SEM images of (a,b) PTFE-0.2 membranes before and after AGMD operation and (c,d) NF-PES-8 membranes before and after AGMD operation in the presence of salt and BSA for 48 hours.

For the NF-coated PES membranes, we observe only very weak coverage by BSA on most of the membrane area and no crack formation (Figure S 5 d). However, more BSA seemed to adsorb in areas where the NF layer spans the large PES pores. In addition, shrinkage of this adsorbed layer during drying seems to have damaged the NF coating along the pore edges, possibly due to stress concentration at the edges during the drying process (Figure S 5 d). This should not be a problem during continuous operation of the membrane but needs to be considered for the case of intermittent operation with complete drying in between process cycles.

Wetting properties using other low surface tension liquids

Advancing and receding contact angles were determined on PE, PTFE, and nanofilament-coated membranes using an ethanol/water combination. Ethanol mixtures of 20 vol% (surface tension - 38 mN/m) and 30 vol% (surface tension - 33 mN/m) were prepared. For reduced surface tension, as expected, both PE and PTFE membranes showed a loss of liquid repellency. However, nanofilament-coated membranes also showed a loss in hydrophobicity as RCA decreased to ~102 ^{o} for 30 vol% ethanol.

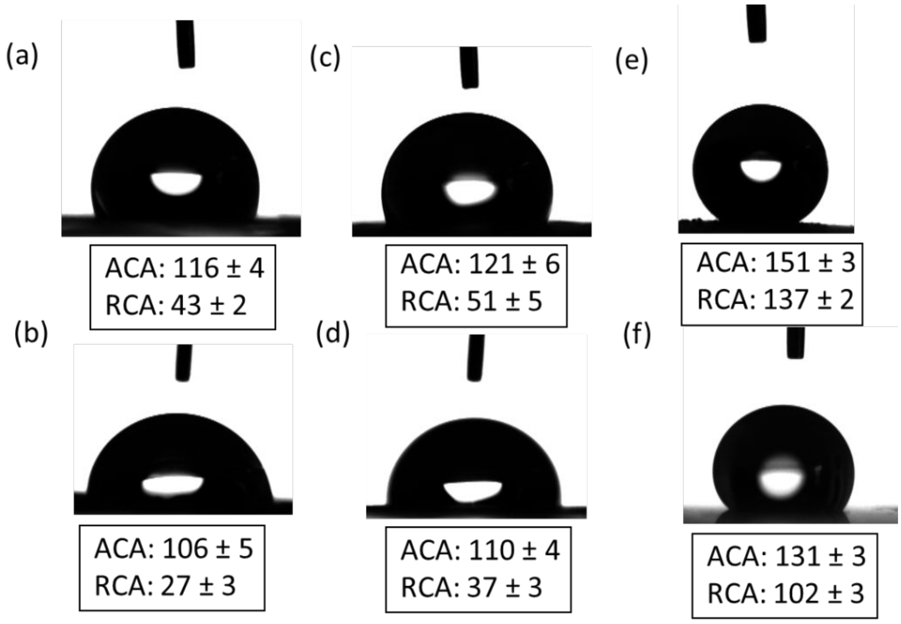

Figure S 6: Advancing and receding contact angles (ACA and RCA) for 20 vol% ethanol mixture (surface tension of 38 mN/m) (a) PE (c) PTFE (e) NF-PES membranes. ACA and RCA for 30 vol% ethanol mixture (surface tension of 33 mN/m) (b) PE (d) PTFE (f) NF-PES membranes.

References

1. Chi Yang, Xue Li, Jack Gilron, Ding Kong, Yong Yin, Yoram Oren, Charles Linder and Tao He, CF4 plasma-modified superhydrophobic PVDF membranes for direct contact membrane distillation. Journal of Membrane Science, 2014. **456**: p. 155-161.

2. Sebastian Leaper, Ahmed Karim, Tarek Allah and Patricia Gorgojo, Air-gap membrane distillation as a one-step process for textile wastewater treatment. Chemical Engineering Journal, 2018. **360**: p. 1330-1340.

3. Mourad Laqbaqbi, García-Payo, Mohamed Khayet, Jauad Kharraz and Mehdi Chaouch, Application of direct contact membrane distillation for textile wastewater treatment and fouling study. Separation and Purification Technology, 2019. **209**: p. 815-825.

4. Mohamed Elmarghany, Ahmed Shazly, Mohamed Salem, Mohamed Sabry, and Norhan Nady, Thermal analysis evaluation of direct contact membrane distillation system. Case Studies in Thermal Engineering, 2019. **13**: p. 100377.

5. Giuseppe Grasso, Francesco Galiano, M. Yoo, R. Mancuso, Hobum Park, Bartolo Gabriele, A. Figoli and Enrico Drioli, Development of graphene-PVDF composite membranes for membrane distillation. Journal of Membrane Science, 2020. **604**: p. 118017.

6. Chunlei Su, Thomas Horseman, Hongbin Cao, Kofi Christie, Yuping Li and Shihong Lin, Robust Superhydrophobic Membrane for Membrane Distillation with Excellent Scaling Resistance. Environ. Sci. Technol, 2019. **53**: p. 11801-11809.

7. Kharraz, J. A. *et al.* Macro-corrugated and nano-patterned hierarchically structured superomniphobic membrane for treatment of low surface tension oily wastewater by membrane distillation. *Water Res.* **174**, 115600 (2020).

8. Lee, C.-K., Park, C., Woo, Y. C., Choi, J.-S. & Kim, J.-O. A pilot study of spiral-wound air gap membrane distillation process and its energy efficiency analysis. *Chemosphere* **239**, 124696 (2020).

9. Kebria, M. R. S., Rahimpour, A., Bakeri, G. & Abedini, R. Experimental and theoretical investigation of thin ZIF-8/chitosan coated layer on air gap membrane distillation performance of PVDF membrane. *Desalination* **450**, 21-32 (2019).

10. Alpatova, A., Alsaadi, A. S., Alharthi, M., Lee, J. G. & Ghaffour, N. Co-axial hollow fiber module for air gap membrane distillation. *J. Membr. Sci.* **578**, 172-182 (2019).

11. Abdel-Karim, A. *et al.* PVDF membranes containing reduced graphene oxide: Effect of degree of reduction on membrane distillation performance. *Desalination* **452**, 196-207 (2019).

12. Andrés-Mañas, J. A., Ruiz-Aguirre, A., Acién, F. G. & Zaragoza, G. Performance increase of membrane distillation pilot scale modules operating in vacuum-enhanced air-gap configuration. *Desalination* **475**, 114202 (2020).

13. Alkhudhiri A, Bin Darwish N, Hakami MW, Abdullah A, Alsadun A, Abu Homod H. Boron Removal by Membrane Distillation: A Comparison Study. *Membranes* **10**, 263 (2020).

14. Khayet, M., García-Payo, C. & Matsuura, T. Superhydrophobic nanofibers electrospun by surface segregating fluorinated amphiphilic additive for membrane distillation. *J. Membr. Sci.* **588**, 117215 (2019).

15. Dudchenko, A.V., Haridkar, M., Xin, R., Joshi, S., Wang, R., Sharma, N., Mauter, M.S. Impact of module design on heat transfer in membrane distillation. *J. Membr. Sci.* **601**, 117898 (2020).
16. Youmin Hou, Prexa Shah, Vassilis Constantoudis, Evangelos Gogolides, Michael Kappl and Hans-Jürgen Butt; A super liquid-repellent hierarchical porous membrane for enhanced membrane distillation. Nature Communications, 2023. **Accepted**.
